# Current Advances of Polysaccharide-Based Nanogels and Microgels in Food and Biomedical Sciences

**DOI:** 10.3390/polym14040813

**Published:** 2022-02-20

**Authors:** Aristeidis Papagiannopoulos, Konstantinos Sotiropoulos

**Affiliations:** 1 Theoretical and Physical Chemistry Institute, National Hellenic Research Foundation, 48 Vassileos Constantinou Avenue, 11635 Athens, Greece; 2 Hyad Mike, Nutritional Supplements Manufacturing Company, Gennadiou 1-5, 12131 Athens, Greece; kwstas_sw@hotmail.com

**Keywords:** polysaccharides, nanogels, microgels, proteins, drug delivery, nutraceutical delivery, emulsion stabilization

## Abstract

Polysaccharides are natural polymers with hydrophilic, biocompatible and biodegradable characteristics and have many opportunities in the food and pharmaceutical sectors. This review focuses on the field of nano and microstructures whose internal structure is based on networked polysaccharide chains in 3D i.e., polysaccharide nanogels (NGs) and microgels (MGs). As it is observed the number of articles on NGs and MGs in peer reviewed scientific journals has been increasing over the last two decades. At the same time, the relative contribution of polysaccharides in this field is gaining place. This review focuses on the different applied methods for the fabrication of a variety of polysaccharide-based NGs and MGs and aims to highlight the recent advances on the subject and present their potentials and properties with regards to their integration in aspects of medicinal and food sciences. The presentation of the recent advances in the application of polysaccharide NGs and MGs is divided in materials with potential as emulsion stabilizers and materials with potential as carriers of bioactives. For applications in the medical sector the division is based on the fabrication processes and includes self-assembled, electrostatically complexed/ionically crosslinked and chemically crosslinked NGs and MGs. It is concluded that many advances are expected in the application of these polysaccharide-based materials and in particular as nutrient-loaded emulsion stabilizers, viscosity modifiers and co-assembled structures in combination with proteins.

## 1. Introduction

Polysaccharides are naturally derived biopolymers with hydrophilic properties and with polyelectrolyte properties in many instances [1]. As a class of polymers they are considered biocompatible, nontoxic and biodegradable and therefore are very attractive and broadly applicable in food technology [2], biomedical sciences [3] and agrochemical industry [4]. They are versatile macromolecules as they can interact with other molecules either in their original form or conjugated with other molecular or macromolecular entities. Self-assembly and co-assembly for the preparation of biomaterials that include polysaccharides are promising since they do not involve the use of any toxic reactants or solvents [3]. Polysaccharides are very often involved in functional nanostructures for a range of applications such as the development of nanoparticles for drug and nutrient delivery [5], hydrogels for tissue regeneration [6] and films for food packaging [7].

The exciting properties of hydrogels have attracted the interest of the research community for several decades because of their rich morphology [8,9]. Hydrogels provide hydrophilic porous structures of 3D polymeric networks that may deliver drugs, proteins and growth factors to cells and tissues [10,11] and can be also used to accommodate cells for proliferation and tissue growth [12]. These properties are retained in the micro- or nano-level and are therefore suitable as micro- and nano-particles in applications that include nanodelivery of drugs, nutrients and proteins and bioimaging. Nanoscopic or microscopic hydrogels i.e., nanogels (NGs) or microgels (MGs) can be considered as an alternative to compact nanoparticles and microparticles as they have a permeable interior that can encapsulate and release bioactive compounds, respond to external stimuli by swelling/deswelling transitions and interact with other molecules by electrostatic, hydrophobic and other kinds of interactions.

Various polysaccharides have been used by researchers for the fabrication of NGs/MGs either solely or combined for the formation of complexes. Among others, NGs/MGs based on Alginate, Hyaluronic acid, Pectin, Dextran and Chondroitin sulfate are presented in the current review. Chitosan, a polysaccharide that has been extensively studied due to its positive charge that makes it ideal for polyelectrolyte complexation with different anionic polysaccharides is also analyzed for both medicinal and food applications. These materials are well-known in these relevant fields as they are broadly used for decades due to their properties.

In this review the current research on NGs and MGs that are made by polysaccharides is presented with focus on their applications and potential in the food sector and in the field of biomedical sciences. Recent literature is reviewed so that the increasing interest on polysaccharide NGs and MGs is highlighted. The section of food science is divided in two subsections based on NGs/MGs applications regarding emulsion stabilizers and carriers of bioactive agents. For medical applications the division is made according to the preparation methods of the NGs and MGs so that recent developments on self-assembly, electrostatic complexation/ionic crosslinking and chemical bonding are presented. This review intends to motivate the use of polysaccharides for the preparation of NGs and MGs taking advantage of the knowledge gained from both the fields of food and medical science.

## 2. Motivation for Investigations on Polysaccharide NGs and MGs

**Figure 1:**
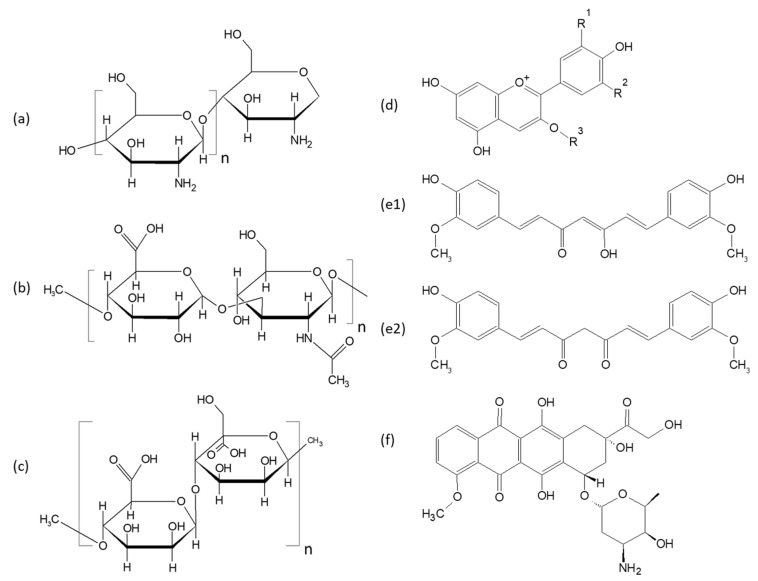
Chemical structure of chitosan (**a**), hyaluronic acid (**b**), alginate (**c**), anthocyanins (general structure) (**d**), curcumin in the enol (**e1**) and keto (**e2**) form and (**f**) doxorubicin. In d R^1^ and R^2^ can be H, OH or OCH_3_ and R^3^ can be 3-O-glucoside.

The hydrophilic nature of polysaccharides, their possibility to carry chargeable units and the potential for chemical modifications allows them to create functional nanostructures and microstructures that may also act as carriers of active molecules. Figure 1 presents the chemical structure of several polysaccharides that are very often used in food science and pharmaceutics for the preparation of NGs and MGs and of some active molecular compounds that are customarily encapsulated in the aforementioned structures.

**Figure 2:**
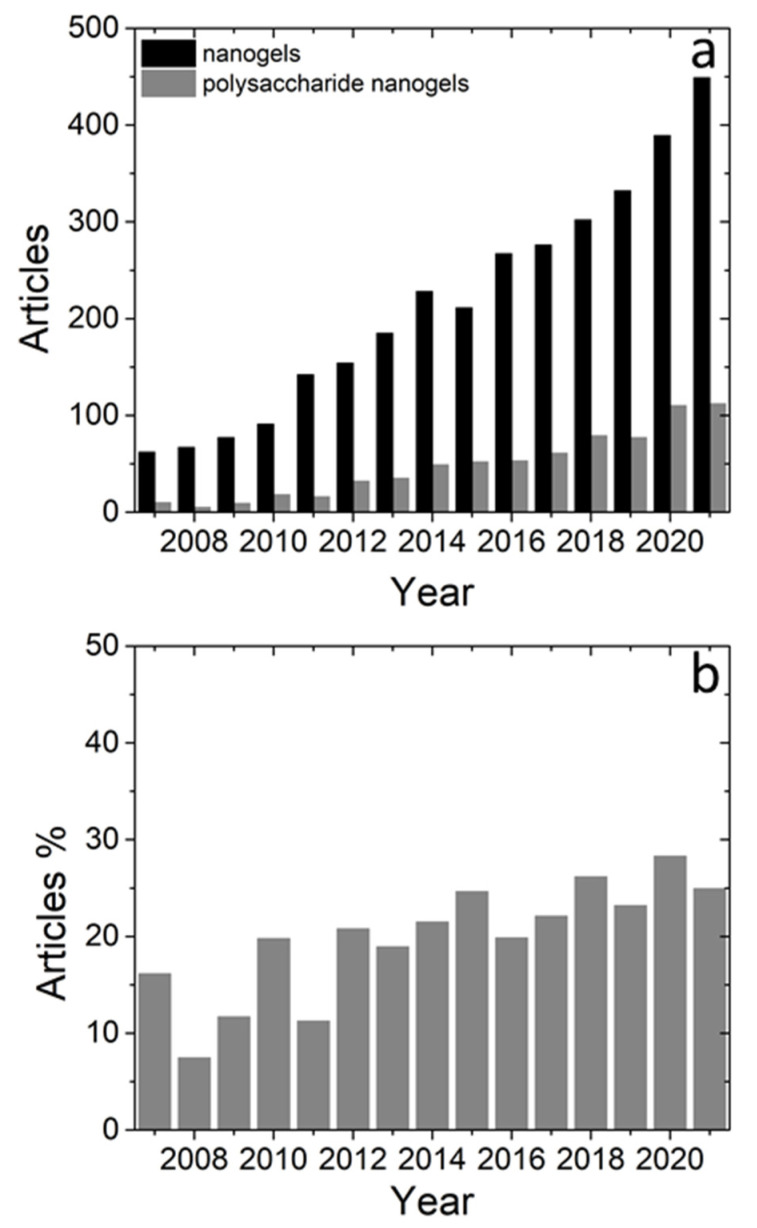
(**a**) Published articles in peer-reviewed journals on NGs (black) and polysaccharide-based NGs (grey). (**b**) Ratio of articles on polysaccharide-based NGs over articles on NGs (source: Scopus).

**Figure 3:**
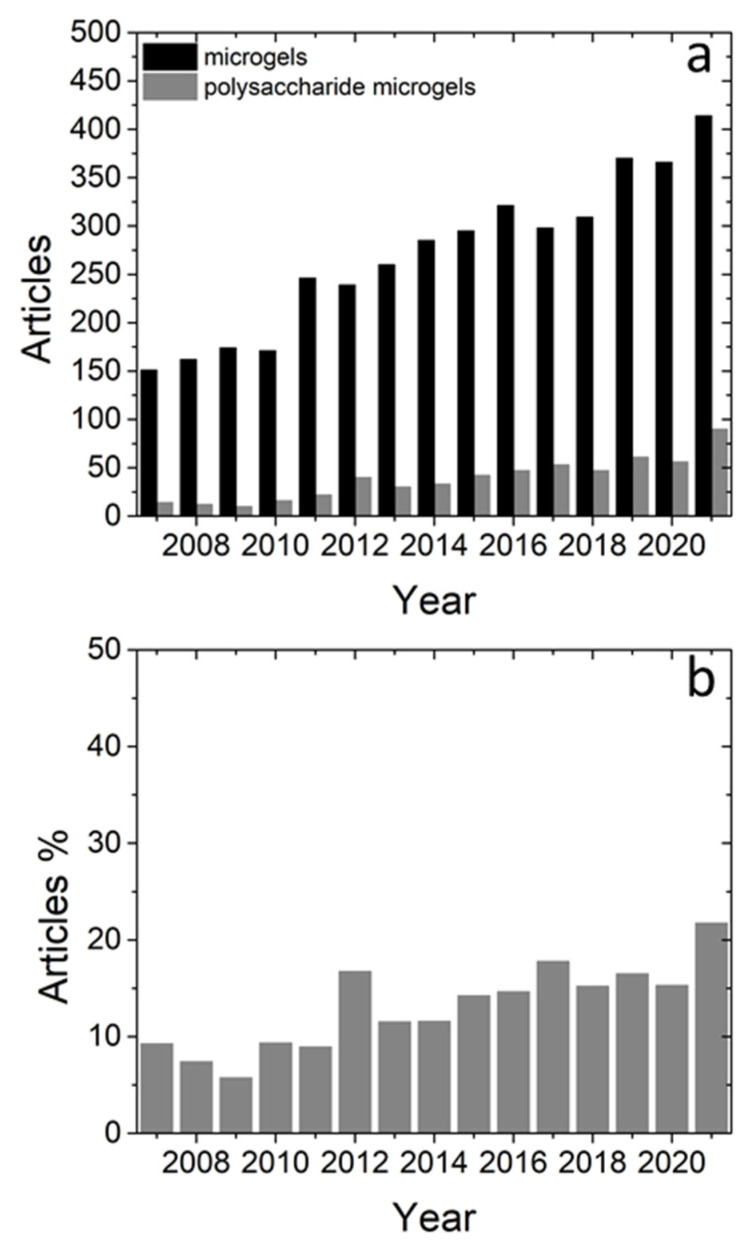
(**a**) Published articles in peer-reviewed journals on MGs (black) and polysaccharide-based MGs (grey). (**b**) Ratio of articles on polysaccharide-based MGs over articles on MGs (source: Scopus).

The research on polysaccharide NGs and MGs has been growing in the last 15 years as it can be seen from the annual production of relevant articles in Scopus. The search was based on articles with the term “nanogel” (Figure 2a) or “microgel” (Figure 3a) in their title, abstract or keyword list. In more detail, articles per year on NGs increased almost 9 times, while articles per year on MGs increased systematically about 3 times between 2007 and 2021. In order to capture the trend in articles’ production related to NGs and MGs based on polysaccharides the previous search was narrowed down to the instances that contained one or more of the terms, “chitosan”, “hyaluronic”, “alginate”, “cellulose”, “starch”, “pectin”, “chondroitin sulfate”, “dextran” and “xanthan” which represent very commonly used polysaccharides in the field of NGs and MGs. The outcome of this search is referred to as “polysaccharide nanogels” and “polysaccharide microgels” and is presented in Figure 1 and Figure 2 respectively.

From Figure 1a and Figure 2a it can be seen that research on polysaccharide NGs and MGs follows the increasing trend of NGs and MGs. The percentage of polysaccharide NGs over the NGs articles (Figure 2b) shows that polysaccharide NGs are a significant part of the NGs field. In addition, this relative contribution appears to gain space within the NG field as it has risen from about 10% up to about 25–30%. In the case of MGs, the relative contribution is again significant and rises from about 8% to about 20% (Figure 3b). In conclusion, the current research in polysaccharide NGs and MGs and its importance in the field of NGs and MGs is reflected in the number of articles published and it is also expected to rise in the next years. 

## 3. Polysaccharide NGs and MGs Applications in the Food Industry

### 3.1. NGs and MGs as Stabilizers in Edible Emulsions

NGs and MGs have been extensively studied mainly in the last decades with regards to their potential use in a variety of applications in the food industry [13,14]. Regarding the food science NGs and MGs have drawn attention as novel emulsion stabilizers mainly in oil-in-water food emulsions. A number of ingredients (whey protein, pectin, gum arabic, gelatin, etc.) that have been studied in MGs systems are used broadly in the food industry as surfactants (surface-active agents) in commercial emulsions in order to lower the interfacial tension between oil and water and air and water [15]. The first macromolecule studied for the formation of MG-stabilized emulsions was poly(N-isopropylacrylamide) (PNIPAM) [16]. Subsequently, a number of food-grade ingredients have been analyzed in terms with this application and can be divided into two categories i.e., polysaccharides and biopolymer proteins while also complexes of combined polymer materials have been also investigated [17]. Some examples of MGs and also NGs used as oil-in-water emulsion stabilizers along with their formation mechanism can be found in Table 1.

**Figure 4:**
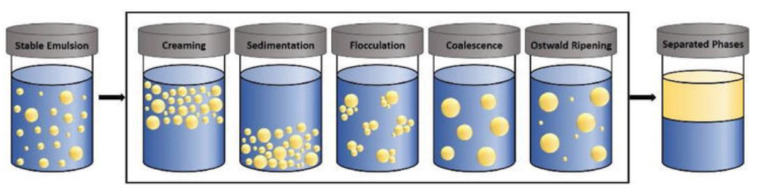
Mechanisms of emulsion destabilization. © 2021 Arantzazu Santamaria-Echart, Isabel P. Fernandes, Samara C. Silva, Stephany C. Rezende, Giovana Colucci, Madalena M. Dias and Maria Filomena Barreiro. Originally published in [28] under the terms of the Creative Commons Attribution 3.0 License. Available from: 10.5772/intechopen.99892.

Long-term stability of emulsions is crucial for food emulsions with long shelf life like mayonnaise and salad dressings is however thermodynamically challenged since the free energy of the two separate phases (oil and water) is lower compared to the free energy of the emulsion system (Figure 4). Demulsification may occur as a result of gravitational separation (creaming/sedimentation), flocculation, coalescence, and Ostwald ripening phenomena [27]. Creaming phenomenon (expressed by the creaming index) occurs as a result of buoyancy leading to droplets rising in the surface, while sedimentation refers to the opposite outcome of denser droplets settling in the bottom of the mixture. Flocculation is the term that is used to describe the formation of droplets’ aggregates while coalescence is the formation of bigger droplets due to merging of separate droplets. Ostwald ripening is attributed to the difference in the solubility of droplets of different sizes leading to larger droplets growing over time at the expense of smaller ones [28].

**Figure 5:**
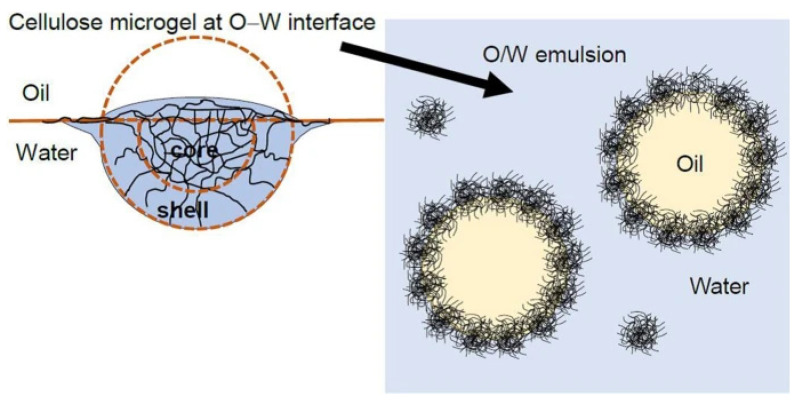
Ellulose microgel at the oil-water surface and oil droplets in water protected by microgel particles. Reprinted from [32].

Emulsions stabilized by solid particles partially wetted by both the aqueous and the oil phase are called Pickering emulsions as they were named after S.U. Pickering who described the phenomenon in 1907. Pickering emulsions have gained attraction in the food industry as an alternative to commonly used artificial surfactants providing stable emulsions with use of food-grade consumer-friendly materials [29]. In general, the type of formed Pickering emulsions is affected by wettability, a characteristic factor dependent on the hydrophobicity of the solid particles, and is indicated by the oil–water interface contact angle. Oil-in-water emulsions are generally stabilized by hydrophilic particles with contact angles θ in the range of 15° < θ < 90°, while water-in-oil emulsions are stabilized by hydrophobic particles with 90° < θ < 165° contact angle range [30]. With respect to their name, MG-based emulsions have been given the name Mickering emulsions (Figure 5). A difference between the two categories of emulsions is that while typical Pickering emulsions are achieved by the use of materials in solid form, the MGs differ in terms of structure as a MG particle consists of a crosslinked network of polymer chains [31]. 

Chitosan (Chit) is one of the most studied polysaccharides for the fabrication of NGs in order to stabilize Pickering emulsions [19]. Since it is highly hydrophilic it cannot stabilize oil-in-water emulsions by itself. Modification of Chit by the formation of amide bonds with stearic acid (via carboxylic acid groups of stearic acid and free amino groups of Chit) was studied in order to stabilize sunflower oil-in-water Pickering emulsions. Measurements of creaming index and droplet size range, at different Chit-stearic acid ratios and fixed oil-to-nanogel ratio (20:1) showed that after 7 days of storage, better stabilization of the emulsions was achieved at higher presence of stearic acid in the nanogels (0.5:1 ratio of stearic acid:Chit). A possible explanation for the effect was the reduced hydrophilicity and positive charges of Chit due to the increase in amide linkages as a consequence of the increase of the stearic acid content. Moreover, better stabilizing performance of the NGs was met at alkaline pH (between 8 and 10). This could be attributed to the neutralization of Chit’s positive charges at elevated pH that led to a better coverage of the surface of oil droplets due to the reduction of electrostatic repulsions [18]. Chit-stearic acid nanogels have also been studied for the stabilization of Pickering emulsions and incorporation of clove essential oil for the formation of a novel type of mayonnaise [33].

Chit hydrochloride/carboxymethyl starch complex (ChitHCl/CMS) NGs have been studied for the stabilization of Pickering emulsions. Amide linkage between the two materials was induced by an EDC-mediated reaction. The smallest particle size (380 nm) was reached by ChitHCl/CMS nanogels in a ratio 2:1 and was selected as more suitable for stabilization. NGs showcased contact angle of nearly 90°, suggesting capability of stabilizing the formed emulsion. ChitHCl/CMS were used in a fixed 1.5% concentration for the preparation of samples with different oil phase fractions. After 3 months, the Pickering emulsions with an oil phase fraction φ = 0.5 were found to be stable. The enhanced stability compared to the prepared emulsions with lower oil phase fraction and was attributed by the authors to the high viscosity of the emulsion and the network architecture of the densely packed oil droplets [21].

In another study MGs were formed by conjugating whey protein isolate (WPI) with dextran (Dex) via Mailard reaction (bonding between amino groups of the protein and residual sugars of the polysaccharide) and were subsequently used for the stabilization of emulsions. They provided delayed interfacial gastric proteolysis indicating the potential use of such MGs for the fabrication of gastric-stable Pickering emulsions delivery systems. MG particles were obtained by top-down method and had hydrodynamic diameters of 136–146 nm. Oil-in-water (20% wt MCT oil) emulsions were prepared with high pressure homogenization and MG particles (1% wt protein concentration) with low degree of conjugation (10%) successfully stabilized the emulsions. The long-term stability of the emulsions was attributed to the particle stabilization provided by the MGs [23]. 

Among other factors, the effect of MGs’ preparation method (building-up vs. breaking-down) on the emulsifying ability of polysaccharide-based MGs has been studied. Results showed that agar and curdlan MGs emulsified soybean oil emulsions with similar oil droplet size proving that the preparation method did not affect the property. Creaming stability on the other hand appeared to be dependent on the microgelation method. Slower initial creaming rate was observed for agar MGs-stabilized emulsions when MGs were fabricated by the building-up method in comparison to MGs prepared by the breaking-down method. The opposite effect was found for the curdlan-based emulsions [34]. Besides the application of MGs as stabilizers for Pickering emulsions, the potential use of such materials in Pickering foams has also been proposed. Nanocellulose MGs which were fabricated with a bottom-up technique by electrostatic assembly between nisin and 2,2,6,6-tetramethyl piperidine-1-oxyl-oxidized cellulose nanocrystals (TOCNC) have been analyzed by transmission electron microscopy (TEM). They showed dendritic morphology for concentration of nisin 0.03% and 0.06% wt and enhanced Pickering foam forming property and stability for 0.06 % nisin. The foam stabilizing effect of the MGs was indicated by the remarkable decrease of interfacial tension between the aqueous dispersion and air from 71.5 to 49.4 mN/m for the 0.06% wt nisin samples [35]. Xin lit et al. [36] studied foams stabilized by combinations of egg white protein (EWP), a common foaming agent in edible foams, and EWP MGs at various proportions including EWPM-only stabilized foams. Presented data showed that EWPM Pickering foams were more stable during processing treatment applied (microwave-heating) and addition of EWPM generally enhanced foam stability against bubble disproportionation while EWP offered higher foamability compared to foams stabilized by MGs.

A significant limitation in the typical Pickering emulsions lays on the demulsification occurring at specific pH values. Various food emulsion formulations may often require a desired acidic pH-range (due to active ingredients’ properties, use of preservatives or other reasons) that cannot always be achieved stability-wise in the desired extend with the use of solid particles. Thus, polysaccharide-based MGs can constitute a significant alternative. Long-term stability investigation of emulsions stabilized by pectin-based MGs revealed that formation of agglomerates and creaming was observed in the samples when the pH was adjusted to 2 and 3. Based on the results it was suggested that charged MGs could not effectively stabilize emulsions at pH lower than their pKa [37].

Due to their remarkable properties MGs can also be used as rheology/viscosity modifiers. Whey protein MGs dispersed in a Newtonian corn syrup and in a non-Newtonian Xanthan gum complex fluid appeared to increase the viscosity when added in a continuum of low viscosity. On the other hand, in more viscous fluids an opposite effect was observed as the MGs acted as thinning agents. At the same time a dependence on the MGs’ rigidity was observed. Increased high shear rate viscosity of solutions was achieved by MGs of higher elastic modulus [38]. Further investigation of polysaccharide-based MGs, effect on type and level of crosslinking, preparation method, level of internal structure’s inhomogeneity [39] and characteristics of various media could offer better understanding as for their role in this application.

### 3.2. Polysaccharide NGs and MGs as Delivery Systems of Nutrients

Nano and microcarriers for encapsulated substances have been a field of interest for research in the pharmaceutical industry for several decades due to their protective action for the labile active ingredients and for their ability for targeted release. In food science and especially in nutraceuticals, encapsulation can significantly enhance the stability and bioavailability of various active ingredients such as vitamins, minerals (zinc, magnesium, calcium et al.), phytochemicals and more specifically polyphenols (anthocyanins, curcumin, quercetin et al.), enzymes, probiotics and polyunsaturated fatty acids [40]. These molecular agents are found to degrade easily due to temperature and pH changes, light, oxidation and low water solubility in their free form [41,42].

In general, delivery systems for pharmaceuticals and nutraceuticals must be based on the use of non-toxic, safe and absorbable ingredients and should have satisfactory loading capacity, protection of the loaded ingredients against possible degradation by e.g., pH, temperature and metal ions, efficient release rate of the substances, high storage stability, enhanced bioavailability and absorption in the gastrointestinal tract [43]. MGs/NGs seem very promising as encapsulation carriers due to their ability to be stimuli-responsive to a number of external factors such as temperature, pH and light. Several examples of MG and NG carriers, their encapsulated substances and potential applications are shown in Table 2.

Anthocyanins are flavonoids that give the characteristic color (blue, red, purple) to various vegetables and fruits (blueberries, cranberries, grapes and others) and have been a subject of research concerning human health, with potential health benefits ranging from antimicrobial and visual health, to cardiovascular diseases and cancer while they are also used in the food industry as natural colorants [54,55]. As anthocyanins belong to a group of compounds whose stability is challenged by increased pH and temperature, stability enhancement provided by the use of delivery systems could be crucial. For this reason, different encapsulation carriers and techniques have been developed including spray/freeze drying, emulsification, gelation and ultrasonication. MG-encapsulated anthocyanins have been developed and analyzed by researchers with promising results showcasing improved bioavailability, stability and protection from degradation in the upper gastric tract [56,57]. In another study, Tan et al. [44] presented novel MGs consisting of polyelectrolyte complexes (PECs) of chondroitin sulfate (CS) and Chit loaded with anthocyanins and incorporated in alginate (Alg) MGs synthesized with the emulsification/internal gelation method. The presence of PECs led to highly rigid MGs during freeze drying and significant reconstitution capacity upon rehydration. 

Ji analyzed the behavior of anthocyanins encapsulated in porous modified starch microgels [58]. In the study, corn starch was modified through TEMPO-oxidation and hydrolyzed by glycoamylase. STMP (trisodium phosphate) was used as a cross-linker for the fabrication of MGs and comparative results between porous and oxidized MGs were obtained. Porous starch MGs were considered more suitable for the encapsulation of anthocyanins due to the adsorption mechanism of anthocyanins via electrostatic interactions between their cationic groups and the charge density of the microgel surface. In addition, the comparison of free anthocyanins and anthocyanins encapsulated in starch porous MGs in terms of storage stability highlighted significant improvement in favor of the porous MG. Approximately 31% residual rate of encapsulated anthocyanins in porous MGs, 18% in oxidized MGs and 8% of free anthocyanins were observed after 30 days at 37 °C.

Calcium-Alg MG particles have been prepared for the encapsulation of water-insoluble polyphenols and β-carotene. Separate mixtures containing encapsulated β-carotene, rutin, tiliroside and curcumin and 2% wt Alg were introduced to a jet homogenizer in order to produce the MG particles. Results showed high levels of loading efficiency especially for particles with rutin and tiliroside (>50%), while dependence on the size and possibly the surface charge density of the encapsulated particles was observed [45]. Calcium-Alg MGs have also been investigated as novel delivery systems of garlic flavor. Allyl methyl disulfide (AMDS), a lipophilic compound of garlic was introduced in an oil-in-water emulsion and subsequently mixed with aqueous sodium Alg solution. The MGs were eventually incubated in a CaCl_2_ solution. Mean particle diameters of the fabricated MGs fell in the range of 270–410 nm with monomodal particle size distribution. Simulated cooking conditions were applied in order to test the control release of the flavor. After boiling in 30 min MGs were found to be intact to a large extend which indicated heat-stability. Flavor retention capacity was measured in terms of the time when the 50% of the AMDS particles were released. For the MGs this time was 12.6 min while for AMDs loaded in oil-in-water emulsions was 3.2 min [59].

Chit, whose properties include the unique cationic charge, is abundant in nature and is possibly the most studied polysaccharide for the formation of NGs and MGs as encapsulation carriers of active ingredients. A recent study presented high molecular weight Chit-based NGs for the encapsulation of resveratrol, a polyphenol naturally found in red wine and the skin of red grapes as well as in other sources. Resveratrol is known for its antioxidant properties while research has also focused on potential anti-inflammatory and antiviral activity. NGs were prepared by ionic-gelation method using sodium tripolyphosphate as crosslinker [46]. Encapsulation efficiency was found to reach 60%. In another study, polyanion citrate was used for the crosslinking of Chit-based NGs via bonding between the amino groups of Chit and the carboxyl groups of citrate. The NGs were tested with regards to the improvement of green tea extract antioxidant activity. The maximum encapsulation efficiency (~70%) was reported in Chit:green tea ratio of 1:0.5. FTIR data indicated hydrogen bonding interactions between hydroxyl groups of Chit and the polyphehnolic groups of green tea. Scavenging activity of the green tea loaded nanogels was found to increase in comparison to that of free green tea [47]. 

**Figure 6:**
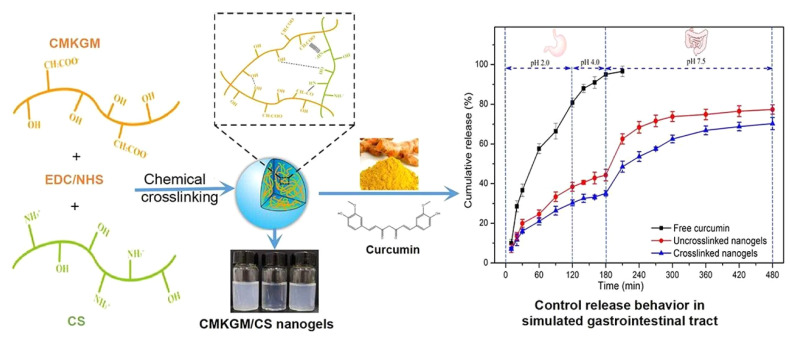
Ormation of cross-linked CMKG/Chit nanogels and release profile of free curcumin and encapsulated curmumin in ucrosslinked/crosslinked nanogels under simulated gastrointestinal conditions. CMKG: carboxymethyl konjac glucomannan, CS: Chit. Reprinted from [49], Copyright (2021), with permission from Elsevier.

Konjac glucomannan is another polysaccharide that has been studied by researchers for the formation of NG complexes with Chit. Carboxymethyl konjac glucomannan (CMKG)/Chit NGs were prepared with the covalent crosslinking method and initiated crosslinking (1-ethyl-3-(3-dimethylaminopropyl)/N-hydroxysuccinimide) (EDC/NHS) was additionally applied in order to study the properties of crosslinked and uncrosslinked NGs (Figure 6). FT-IR and XRD measurements showcased covalent (amide linkage) and non-covalent bonding (hydrogen bonds/electrostatic interactions) between CMKG and Chit as proposed by the authors. Comparative results revealed that the crosslinking with EDC/NHS reduced the zeta potential of the nanogels and increased the encapsulation efficiency while no differentiation of average particle size and morphology of the NGs was found. The prepared NGs were further explored for the controlled release of curcumin under simulated gastrointestinal conditions with promising results [49]. It is worth mentioning that the average particle size of the NGs increased upon the loading of the substance as reported in other studies [47,49]. Regarding the release profile of encapsulated curcumin in the NGs during 8 h period, slower release of curcumin encapsulated in crosslinked NGs was reported suggesting improved retention capacity. Additionally, release of curcumin was found to be pH-dependent (Rate pH 4.0 < Rate pH 2.0 < Rate pH 7.5) (Figure 6).

Oral administration of probiotics has gained increasing attraction as beneficiary of people’s health by modifying gut mircobiota. Probiotics however have been found to be extremely sensitive to heat and moisture and also threatened by the acidic environment of the gastric fluids. Thus, the protection of such ingredients during processing and digestion can lead to significantly improved final products. MGs fit as size to encapsulate probiotics whose microbial cells fall in the range of 1 to 10 μm [60]. A recent study focused on the encapsulation of *Lactobacillus casei* and *Lactobacillus rhamnosus* strains in pectin MGs. Loaded MG particles were prepared by ionotropic gelation with the appliance of CaCl_2_ as crosslinking agent. The preparation method led to notable encapsulation efficiency of up to 96 ± 4%. Addition of prebiotic inulin was found to further enhance the microbial survival in a monitored 42 days storage period [48].

Soy protein/soy polysaccharide complex NGs have been developed by Ding and Yao as encapsulation carriers of folic acid, which is the synthetic form of folate (vitamin B9). The preparation method constituted by mixing folic acid with the protein and the polysaccharide at pH 7.4, lowering of the pH to 4.0 and then applying high-pressure homogenization and heat treatment. Dynamic light scattering (DLS) measurements revealed polysaccharide surface of the nanogels (estimated layer of 16 nm), making them dispersible at acidic pH values. UV irradation caused remarkably low degradation degree (17%) of loaded folic acid suggesting that nanogels can effectively protect folic acid by photodegradation, a common factor of folic acid’s destabilization [50].

Pickering emulsions have also been studied as delivery systems for hydrophilic as well as lipophilic bioactives showcasing significant properties including enhanced physical and oxidative stability, compatibility and protection of bioactive compounds [61]. Crosslinked Chit MGs have been applied as stabilizers of high internal phase emulsions, a type of concentrated systems with a volume fraction of internal or disperse phase higher than 74%. Genipin was used for the crosslinking of the Chit MGs. Stable emulsions with an internal phase volume of 80% (consisting of dodecane) were formed with the addition of 0.1% wt. of various MGs with different Chit molecular weights (50:100:150 KDa) and mass ratios of Chit and genipin (2:1. 5:1, 10:1. 20:1). The systems were further studied for the encapsulation of β-carotene. Dispersion of β-carotene in the oil phase, was followed by mixing with aqueous MG suspensions. Contents of encapsulated β-carotene were found to be up to 2% wt with 0.1% wt of emulsifiers. In addition, results showed enhanced stability of β-carotene against harmful factors for the substance such as ultraviolet irradiation, high temperature thermal treatment, interaction with metal ions (iron) and hydrogen peroxide [20].

It is worth mentioning that NGs have also been studied as applicants for the development of Pickering emulsions suitable for pharmaceutical applications. So far, mainly protein-based NGs have been chosen by researchers. Casein NGs prepared by crosslinking of casein with glutaraldehyde have been successfully used for the fabrication of Pickering high internal phase emulsions and the subsequent encapsulation of hydrophobic drug indomethacin (IDM) in the oil phase [62]. Research of polysaccharide-based MGs/NGs in this field could be expected in the future.

## 4. Medical Applications of Polysaccharide NGs and MGs

Nanoparticles of polymers are widely used in the delivery of drugs with anticancer activity in order to provide sustained and targeted transport [63]. Nanoparticles based on biopolymers are nontoxic, biocompatible and biodegradable [64] and therefore attractive for the aforementioned applications. Recent literature offers many investigations on the use of polysaccharide NGs (Table 3) and MGs (Table 4) for applications in pharmaceutical and biomedical sciences. 

### 4.1. Self-Assembled Polysaccharide NGs

**Figure 7:**
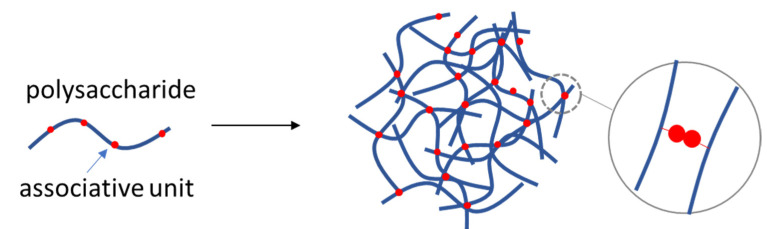
Self-assembly of a polysaccharide in aqueous medium driven by associative units along its backbone.

Self-assembly is at the center of interest as it is essentially free of using toxic organic solvents or chemical reactions. However, as polysaccharides are normally highly hydrophilic and very often chargeable in polar media modifications are needed to induce strong self-assembling properties. Associative units on the backbone of a polysaccharide as for example hydrophobic groups induce self-assembly properties in aqueous media (Figure 7). Hyaluronic acid (HA) was modified by side chains of di(ethylene glycol) methyl ether methacrylate (DEGMA) and 6-bromo-4-hydroxymethyl-7-coumarinyl methacrylate (CMA) monomers. CMA monomers were functionalized with a photolabile coumarin derivative [67]. The molar percentage of the CMA monomers in the copolymer could tune the thermoresponsive self-assembly into NGs. The critical aggregation temperature dropped from 32 °C to 27 °C by increasing the molar content from 3% to 5%. Conjugation of coumarin units to the hyaluronan copolymers resulted to photoresponsive properties. The composition of the copolymers was optimized so that UV irradiation could shift the critical association temperature from below to above the body temperature. The antitumoral action of paclitaxel was greatly enhanced by its encapsulation to the NGs as it was proved on CD44+ ovarian cancer cells [67]. 

**Figure 8:**
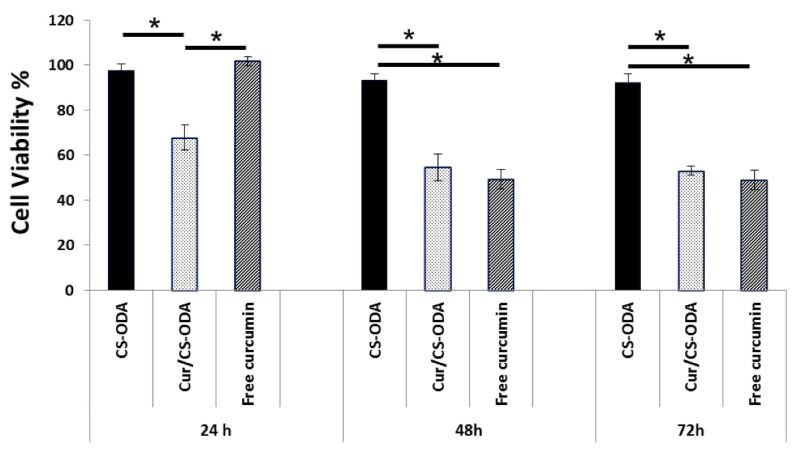
Cytotoxicity of curcumin (Cur)-loaded NGs and free curcumin om MCF-7 cells (human breast cancer). The bars indicate which two groups are being compared. The single asterisks denote statistically significant difference with *p*-value < 0.05. Reprinted from [72], Copyright (2020), with permission from Elsevier.

Chondroitin sulfate (CS) grafted with octadecylamine (ODA) was used to prepare self-assembling NGs [72]. The micellar NGs formed in aqueous media were loaded with curcumin. Several CS/octadecylamine mass ratios were tested and the one that achieved the highest encapsulation efficiency was chosen for in vitro anticancer investigations. Cytotoxicity on human breast cancer cells (MCF-7) was effective for the curcumin-loaded NGs and not for free curcumin within the first 24 h. This was attributed to the higher cellular uptake caused by the NGs. For incubations longer than 48 h the cell viability was the same either for free or encapsulated curcumin (Figure 8). Cellular uptake of curcumin was estimated by fluorescence spectroscopy and indeed it was found that the NGs delivered curcumin more effectively in comparison to the free administered compound. This was attributed to the affinity of CS to the cell membrane as it is an extracellular glycosaminoglycan polysaccharide.

Conjugates between polysaccharides such as CS and prednisolone with glycin linkers are promising for applications in treatments of ulcerative colitis [73] and rheumatoid arthritis [74]. Very recently, Alg-glycyl-prednisolone conjugate NGs have been synthesized in order to overcome the negative effects of high doses of the anti-inflammatory drug [81]. The hydrophobicity of the covalently bonded drug on the polysaccharide backbone led to the self-assembly into NGs in aqueous media. The size of the produced NGs was in the order of 700–800 nm. The rate of the drug release was higher for higher drug contents. In addition, the release was stronger at slightly basic conditions (pH 7.4) in comparison to acidic conditions (pH 6) due to the easier hydrolysis of the ester groups. Pharmacokinetic profiles from the bloodstream of normal rats showed that the NGs achieved prolonged retention of prednisolone (especially for the conjugated drug) in comparison to intravenously injected pure drug. After 7 or 24 h the concentration of the drug in inflamed joints was higher for the loaded NGs in comparison to the drug alone. In addition, in arthritic rats the NGs localized preferably to the joints than to the plasma in contrast to normal rats [82].

Conjugation of CS with the hydrophobic methotrexate was performed to prepare self-assembling NGs [75]. The size of the resulting particles in aqueous solutions ranged from 100 to 400 nm and had an average value of 200 nm. The NGs were tested for their anti-cancer activity in vitro against A549T and Hela tumor cells. The MTT assay showed that there was increased cytotoxicity for both cells contrary to the free drug. In the case of added CS and methotrexate growth of the cells could be observed. CS binds to the transmembrane glycoprotein receptor CD44 [105] and in addition, conjugation of the drug with CS increased its stability leading to the strong enhancement of the cell uptake and effectiveness of the drug [75].

Nanostructures of polysaccharides are currently used for the delivery of proteins to take advantage of the increased stability, permeability and bioavailability of the protein-involving therapies [106]. Cholesterol-bearing pullulan (Pull) was modified by acryloyl groups to prepare NGs for tongue muscle regeneration. Michael addition reaction and the freeze-thaw method was applied to achieve hydrogels that contained crosslinked NGs with porosity up to 70%. The degradation of the hydrogels occurred after 20 days in serum and took longer in PBS. The gels could encapsulate the model protein drug insulin within 60–80 h. The release of insulin was very low in PBS, however in FBS it reached 80% of the total loaded amount within 15 h. This remarkable result was attributed to a protein exchange reaction that is not effective in the absence of other proteins. Mouse myoblast cells survived in the NGs interconnected porous environment for one week and showed normal differentiation characteristics. For in vivo evaluation of muscle regeneration defects in mice tongues were filled with the cell-loaded hydrogels. The regeneration of myofibers, the fundamental structural unit of the skeletal muscle, was significant in the myoblast transplants contained in the hydrogels and also in the myoblast-free hydrogels [85]. In another work, cholesterol-bearing Pull modified by carboxylate groups was used for self-assembled NGs of size about 50 nm. The NGs could effectively transport antigen to the lymph nodes improving the interactions with antigen-presenting cells owing to their anionic charge and were proposed for advanced anticancer immunotherapies [88].

NGs that were prepared from Chit grafted with phenylamine groups were based on host-guest supramolecular interaction in the presence of cucurbit[8]uril. Doxorubicin was encapsulated to the nanostructures by mixing in the system before crosslinking. The NGs could release the loaded drug either by spermine, an overexpressed amine in certain cell types, or by added amantadine. These molecules can replace phenylamine from the cucurbit[8]uril cavity leading to disintegration of the NGs and release of its drug contents. The NGs were able to enter cells and hinder the growth in human lung cancer cell line (A549) which overexpress spermine. On the other hand, they were not as toxic on a human liver cell line (L02) cells where spermine is not overexpressed [77].

### 4.2. Polysaccharide NGs and MGs Formed by Electrostatic Complexation with Other Biopolymers and by Ionic Crosslinking

**Figure 9:**
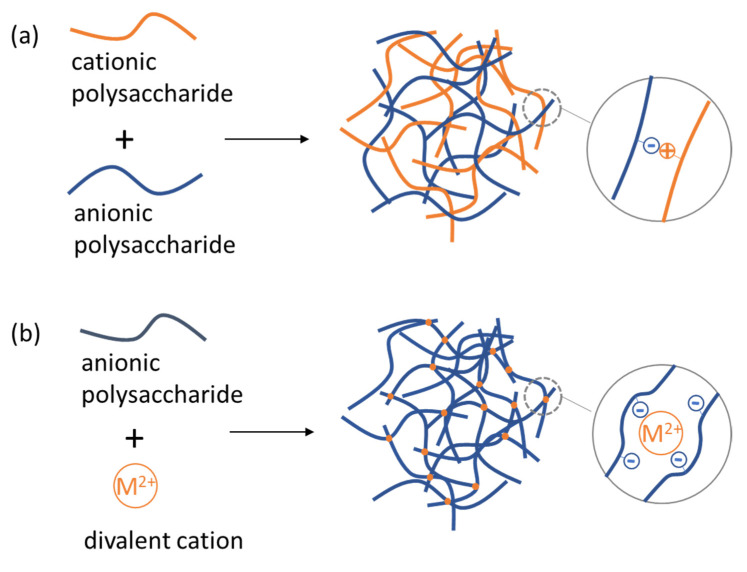
Formation of NGs/MGs by electrostatic complexation of oppositely charged polysaccharides (**a**) and by ionic crosslinking with a divalent metal cation (**b**).

3D networks of polysaccharide chains can be created by electrostatic interactions between the charged groups of polysaccharides and oppositely charged groups of other molecules. As illustrated in Figure 9a an anionic polysaccharide may create electrostatic complexes with a cationic polysaccharide by creating contacts of its negatively charged monomers with the positively charged monomers of the cationic polysaccharide. Multivalent ions can create effective bridges for the crosslinking of a charged polysaccharide. For example, a divalent metal cation acts as an ionic crosslinker for an anionic polysaccharide interacting simultaneously with more than one polysaccharide unit (Figure 9b).

**Figure 10:**
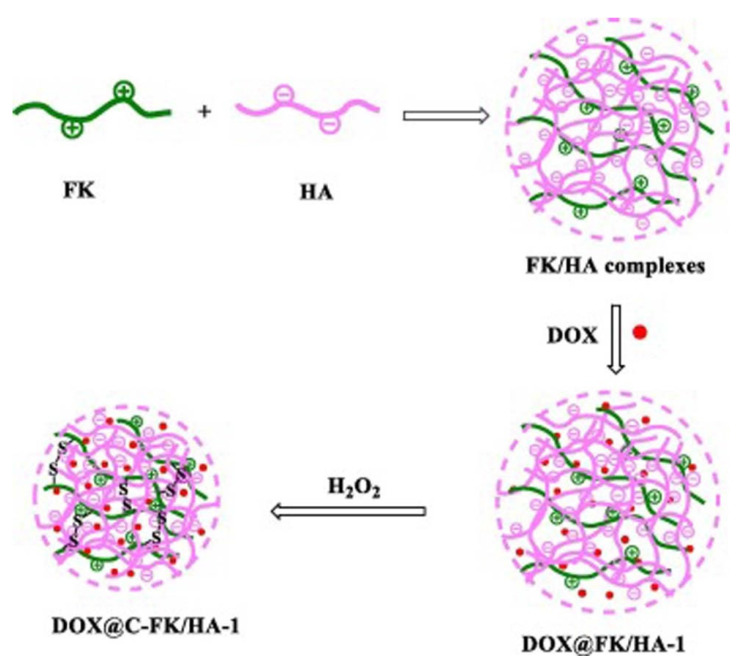
Preparation of the double-crosslinked and DOX-loaded FK/HA NGs. FK: feather keratin, HA: hyaluronic acid, DOX: doxorubicin. Reprinted from [66], Copyright (2020), with permission from Elsevier.

Feather keratin (FK) was used for the dual crosslinking of HA to prepare NGs for DOX delivery [66] as it is illustrated in Figure 10. Ionic crosslinking between the carboxyl groups of HA and the amino groups of keratin-induced pH responsiveness while covalent disulfide bonds with H_2_O_2_ made the NG structure reduction responsive [107]. Increasing the feeding amount of HA led to smaller nanoparticles of hydrodynamic size 300–400 nm with relatively narrow and monomodal distribution. At low amounts of added HA another population in the range of μms was formed. Drug release was tested in phosphate buffered saline pH 7.4 and pH 5.0 acetate buffered solution and in the same buffers with added glutathione to mimic the physiological and tumor conditions respectively. Most of the drug was released in the first 10 h and the phenomenon was sustained up to 30 h. The released amount was higher at pH 5.0 showing the potential of the NGs for targeted delivery to tumors.

Electro-sprayed electrostatic complexes of HA/poly-l-lysine NGs incorporating green fluorescent protein (GFP), DOX and vancomycin (VAN) presented interesting release properties in PBS at physiological temperature. The hydrophobic substance VAN was released with first order kinetics within 4 h while the other hydrophobic drug DOX followed sustained release with zeroth order kinetics for 48 h. GFP did not show significant release for 24 h and was completely released the next 48 h. The release of the protein was caused by the dissociation of the NGs and was not affected by the NG swelling. The NGs could penetrate the membrane of A549 cells and deliver their cargo to the cytoplasm, while free GFP could not be internalized [69]. Pull and fucoidan polyelectrolyte complexation has been reinforced by genipin covalent crosslinking to prepare NGs with size about 160 nm. The NGs were positively charged at acidic pH and were able to bind miRNA. The affinity of fucoidan for P-selectin stimulated the adhesion of the NGs to platelets and endothelial cells making the system promising for treatment of atherothrombosis 

Electrostatic complexation between carboxymethyl cellulose (CMC) and BSA and stabilization by thermal treatment was employed to make NGs for encapsulation of the chemotherapeutic camptothecin (CPT) and the radionuclide ^132^I. These nanoparticles that potentially have both therapeutic and diagnostic action had a spherical shape with diameter 120 nm. Their drug release increased at acidic pH which protected normal tissues from toxic effects. At pH 7.4 the cumulative release of CPT was almost half than the one at pH 5 which was attributed to the weakening of electrostatic interactions between the drug and the NGs [91]. Electrostatic complexation between fibrinogen (Fbg) and HA were stabilized by thermal treatment taking advantage of the thermally-induced intermolecular associations between Fbg molecules [71]. The nanoparticles formed had NG properties and could encapsulate curcumin a well-known nutraceutical [108] also promising for bioimaging applications [109]. In another work, the thermal treatment of BSA within complexes with CS [76] or xanthan [92] resulted in stimuli responsive NG-like nanoparticles that could encapsulate the nutraceutical β-carotene or curcumin respectively. Finally, lysozyme-Dex thermally treated NGs with in situ synthesized gold NPs loaded with DOX were proposed for simultaneous optical cell imaging and cancer treatment [93].

Ionic gelation was used to prepare Chit/laponite NGs. Tripolyphosphate was added to mixtures of laponite and Chit at acidic conditions to achieve crosslinking. The resulting NGs had size in the order of 120 nm in the presence and 130 nm in the absence of laponite. Honey was added to the NGs as model drug. Honey release was characterized by an initial burst and a subsequent sustained release (~5 h). The release was faster at acidic conditions due to the extension of the Chit conformation. The presence of laponite in the nanocomposite nanoparticles was shown to increase the crosslinking density and decrease the release of the drug [78]. Phenolic hydroxyl modified Chit was ionically crosslinked by tripolyphosphate and enzymatically by horseradish peroxidase. The size of the NGs increased from about 250 nm to about 700 nm upon encapsulation of 5-fluorouracil while at the same time their polydispersity also increased. The release of the drug was stronger at acidic pH due to the Chit conformational transition [79].

Alg-based hydrogel nanoparticles were prepared by Alg either modified by mannose for targeting dendritic cells or conjugated with ovalbumin as model antigen for release. The two biopolymers were mixed and crosslinked with CaCl_2_ to prepare NGs of diameter in the order of 300 nm [80]. The strong negative surface charge of the nanoparticles was useful to prevent nonspecific uptake by cells and plasma protein bioadhesion. The release of ovalbumin was much higher at acidic pH in comparison to neutral pH which was attributed to the acid-induced cleavage of Schiff base bond between ovalbumin and Alg. Sustained ovalbumin release was monitored up to 48 h. Internalization of ovalbumin by dendritic cells was much greater when the antigen was loaded on the nanoparticles in comparison to free antigen as it was proved using fluorescent labeling.

Using CaCl_2_ for the gelification of Alg is very common for the preparation of microbeads by dropwise addition of Alg solutions into CaCl_2_ solutions [110]. Alg microspheres in the range of 170–190 μm were produced by flow-focusing in a microfluidic device and dropping in a 10 % *w*/*v* CaCl_2_ aqueous solution [100]. After washing the microspheres were alternatively immersed in solutions of collagen and HA for functionalization with biopolymer multilayers. Myeloma cell lines RPMI 8226 cultures were applied in the produced 3D scaffolds and it was shown that when HA formed the external layer of the functionalized beads cell proliferation was enhanced and resistance to dexamethasone was induced. The produced MGs were proposed as platforms to emulate interactions between the bone marrow extracellular matrix and tumor cells for the investigation of the efficiency of anticancer drugs. Single mesenchymal stem cells encapsulation was achieved in Alg micro-hydrogels that were prepared in a microfluidic device by the formation of beads from a parent hydrogel [101]. Except from CaCl_2_ different metal ions were also used for the ionic crosslinking. These materials were tested for their ability to regulate the bone regeneration by stem cells. It was shown that Ca^2+^ had superior osteoinduction in comparison to Sr^2+^ while on the other hand Sr^2+^ had better ability to inhibit the activity of osteoclasts and hinder bone resorption after regeneration. This resulted in similar bone regeneration efficiencies. Microfluidic technology has been also used for core-shell cell-laden microparticles with a CaCl_2_-crosslinked Alg cell and a collagen core [103]. The MGs could increase the cell viability under the deposition by a 3D-bioprinter. These constructs were proposed for 3D-printed scaffolds for bone regeneration under conditions of reduced cell damage during bioprinting.

**Figure 11:**
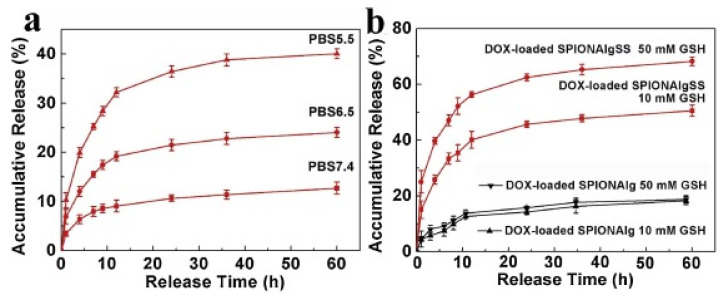
DOX release from DOX-loaded iron-oxide/Alg nanoparticles in (**a**) PBS and (**b**) redox-triggered release by GSH. SPION: superparamagnetic iron oxide nanoparticles, AlgSS: disulfide bond modified Alg. Reprinted from [84], Copyright (2019), with permission from Elsevier.

Alg-based NGs in combination with gadolinium were produced in reverse microemulsion via ionic crosslinking [83]. The NGs were stable for 1–2 months, nontoxic for human neuroblastoma cell and had sizes in the order of 100 nm and negative zeta potential at about −30 mV. The authors achieved to encapsulate hydrophilic drugs and the fluorescent probe rhodamine b which are used for treatment of neurodegenerative diseases and magnetic resonance imaging applications respectively. Another NG system was based on Alg crosslinked by cystamine dihydrochloride to introduce disulfide bonds. The ability of the system for chemotherapy was demonstrated by the targeted delivery of doxorubicin (DOX) to cancer cells without affecting healthy cells. The Alg derivatives were functionalized by superparamagnetic iron oxide nanoparticles so that magnetic resonance imaging was possible [84]. The release of DOX in PBS increased from about 14% at pH 7.4 to about 40% at pH 5.5 for the case of unmodified Alg (Figure 11a). This was due to the protonation of the carboxylic groups at acidic pH that could couple electrostatically with the amine groups of DOX at neutral pH. The release of DOX was not affected by the presence of glutathione (GSH) contrary to the case of disulfide Alg-modified iron oxide NPs where the disulfide bonds could be cleaved and lead to burst release Alg (Figure 11b). Alg has been also used for the delivery of DOX and glycyrrhizin (GL) [85]. The NGs were prepared by crosslinking with Ca^2+^ ions (using CaCl_2_ salt) in solutions containing Alg and drugs. GL was reported to support the ionic crosslinking by forming hydrogen bonds with the polysaccharide. The released amount of DOX was 11% and 47% of the total loaded drug at pH 7.4 and 5.5 respectively owing to the full protonation of the aminogroups at acidic pH. The circulation of the NGs in the bloodstream of rats was prolonged by the presence of GL as the latter hindered their quick uptake by the macrophage phagocytosis [85].

MGs with binary mixtures of Alg and CS or silk fibroin (SF) and tertiary mixtures of the three biopolymers were prepared droplet microfluidics and crosslinked by Ca^2+^ and Zn^2+^ ions in water-in-oil emulsions [96]. The produced MGs had a size in the range of 70–80 μm. The MGs were used to encapsulate nanoparticles for drug delivery. The model nanoparticles were polystyrene nanospheres and BSA-coated polystyrene nanospheres with diameter 100 nm. The release of NPs from the MGs was sustained for several days and could be tuned by the presence of CS and SF. The anionic nature of CS was responsible for the stronger entrapment of BSA-coated NPs and the fibrous nature of SF was attributed the higher steric hinderance of release of both coated and uncoated NPs.

HA (sodium salt) MGs were prepared by physical crosslinking with Fe^3+^ and Gd^3+^ ions resulting from FeCl_3_ and GdCl_3_ salts respectively [94]. The sizes of the MGs ranged from 50 nm to 5 μm. Their zeta potential decrease strongly from about 0 to −30 mV as pH increased from 2 to 4 because of the ionization of the carboxylic acid groups of HA and mildly from −30 to −40 mV when the pH was further increased from 4 to 11 due to the ionization of the hydroxyl groups of HA. Hemocompatibility was confirmed by the low level in the hemolysis of red blood cells (<1%) and increased plasma clotting index (>85 for 1000 μg/mL MGs). The materials were proposed for blood contacting applications and MRI signal enhancers as alternatives to toxic contrast reagents. The MGs were biocompatible at quantities up to 250 μg/mL for HA-Gd and 50 μg/mL for HA-Fe for L929 fibroblast cells.

### 4.3. Chemically Crosslinked Polysaccharide NGs and MGs

**Figure 12:**
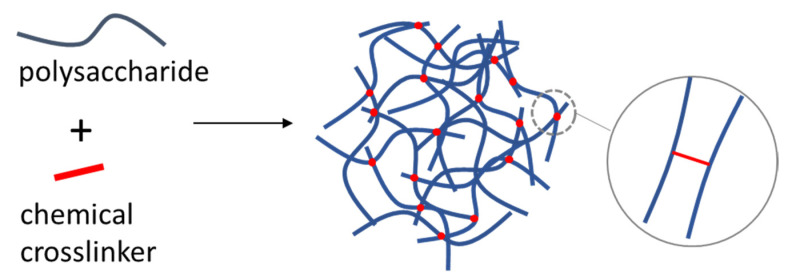
Preparation of a polysaccharide NG or MG with chemical crosslinking.

Chemical crosslinking is an effective method to create stable and well-defined NGs and MGs in a controllable manner (Figure 12). HA NGs were crosslinked by disulfide bonds. This was achieved by methacrylating HA with cystamine [68]. Electrostatically driven encapsulation of an anticancer drug was achieved by using cationic doxodubicin. The NGs were decorated with lactoferrin which is known to associate with the lipoprotein receptor-associated protein 1. This protein is highly expressed in the blood-brain barrier endothelial cells and glioma cells. HA is known for its affinity for CD44 receptors which are overexpressed in malignant tumors. HA has been modified by vinyl groups and cystamine bisacrylamide and used to synthesize hydrogel nanoparticles which were further functionalized by gold clusters. The NGs could selectively accumulate in the interior of the tumors, release DOX and at the same time be fluorescently tracked. The high amount of glutathione in the cancer cells was the trigger for the disassembly of the NGs [70]. 

NGs of κ-carrageenan (κ-Car) and Chit were synthesized by free radical graft copolymerization of acrylamide and sodium acrylate monomers. The aqueous solutions for the one-pot synthesis contained dispersed nitrogen-doped carbon dots. The model drug rivastigmine was attached to the NGs by addition to the NG solutions and complexation. The purpose of the work was to overcome the limitations of Chit imposed by the fast biodegradability, low response in pH and increased solubility in acidic environment and achieve controlled release under intestinal conditions. The degree of swelling increased form gastric (pH 1.2) to intestinal (pH 7.4) conditions due to the electrostatic interactions between the negatively charged groups of Car and acrylate and the neutralization of the charge of Chit. The drug release at pH 7.4 was faster in the case of added carbon dots in comparison to their absence as they increased the hydrophilicity and swelling ratio of the NGs. As expected, the release was weaker at pH 1.2. Carbon dot-containing NGs were biocompatible giving rise to high percentages of primary human fibroblast cells viability up to concentration 62.5 μg/mL [90].

**Figure 13:**
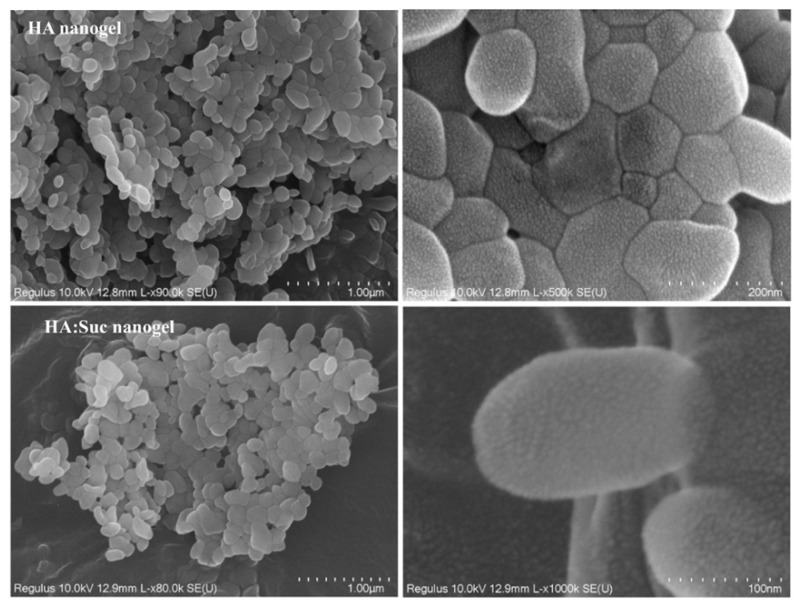
Scanning electron microscopy on Alg and Alg-sucrose hydrogels. Reprinted from [65], Copyright (2019), with permission from Elsevier.

HA porous NGs crosslinked by glycerol diglycidyl ether were prepared by surfactant-free emulsion method [65]. SEM imaging on dried NGs revealed an average size of 150 nm (Figure 13). In water the NGs swelled to about 550 nm. HA/sucrose NGs were also prepared in order to increase the potential of the nanoparticles for targeted delivery to tumor cells. In this case the pore size of the NG (~10 nm) was higher than the one of the pure HA NGs (~4 nm). The increased surface area of the porous nanostructures was proposed as an interesting alternative to nonporous NGs for interaction with bioactive molecules. The hydrophobic anticancer drug 3-((E)-3-(4-hydroxyphenyl)acryloyl)-*2H*-chromen-2-one was loaded either by adsorption or by chemical conjugation to the NGs. It was shown that sustained release over two days could be achieved and that the incorporation of sucrose increased the loaded amount of the anticancer drug.

Conjugation of polysaccharides with phenyl boronic acid and sugar can give rise to dynamic covalent bonding between the chains carrying the different moieties. The boronic-ester crosslinks that stabilized the NGs were effective above pH 7. At lower pH nanoaggregates were not formed and there was a reversible association/dissociation under pH cycles. The NGs were tested for cell studies as their constituents were biocompatible. Indeed, the interaction with kidney cells had negligible cytotoxicity and the NGs were effectively internalized by the cell cytoplasm. NGs did not disassemble sooner than 12 h of incubation [111].

Interpenetrating hydrogels of methacrylated HA and 3-aminophenylboronic acid modified sodium Alg were prepared by crosslinking HA by ultra-violet light and sodium Alg by a dynamic covalent bond between boronic and oxygen in an alkaline environment [102]. Solutions of the two modified polysaccharides were mixed and the hydrogels were formed. Subsequently the hydrogels were passed through a steel mesh and squeezed into MGs. Finally, the MGs were assembled into macroporous hydrogels. These MG-based materials had self-healing properties due to the presence of B-O bonds. Cell migration, ingrowth of cells and blood vessels could be induced and the microstructured hydrogels were promising for tissue regeneration applications.

MGs with antibacterial properties and mechanical stability and pH-responsive release were fabricated for wound dressing applications. A Schiff-base reaction was applied to crosslink carboxymethyl Chit and oxidized carboxymethyl cellulose in emulsion. The MGs were further mixed with carboxymethyl cellulose solutions to prepare composite MG-containing hydrogels. BSA and silver sulfadiazine (AgSD) were loaded as model drugs to the MGs. The gel-MG composites had interesting release profiles as at pH 5.5 and pH 9.5 the release was enhanced. At acidic pH the degradation rate is high and at basic pH the electrostatic repulsion between the polysaccharides leads to high swelling. The composites with loaded AgSD showed good antibacterial activity [97]. Sciff-base reaction has been used also for the crosslinking between CMC and glycol split HA. Porous MGs of size in the order of 4 μm were prepared by the coordination of Zn ions to the reaction. The MGs were biocompatible and could avoid macrophage phagocytosis that was crucial for fast clearance of drug in the lung. In vitro release of BSA was sustained for 24 h and the system was proposed for pulmonary drug delivery [98]. Finally, emulsion polymerization has been used to synthesize Chit MGs (size ~200 μm) reinforced by SiO_2_ nanoparticles for the oral delivery of vitamin-B12 [99]. 

## 5. Conclusions and Future Perspective

Polysaccharides have been extensively used in the food industry and biomedical sciences for decades with versatile applications providing significant benefits being biocompatible, non-toxic, consumer-friendly, cost effective and able to interact efficiently with other biomaterials. As it is proven by the increasing research interest in the last two decades and the recent research advances polysaccharide-based MGs and NGs have potential in various fields of the food industry including their incorporation in nutraceuticals and food products as stabilizers and delivery agents. In medical sciences they are used as drug and protein carriers for the treatment of cancer, healing of rheumatoid arthritis and tissue regeneration. The investigation of these systems as viscosity modifiers and nutrient-loaded emulsion stabilizers in food products is required for industrial production at large scale. Studies on NGs and MGs made from electrostatic complexes between polysaccharides and proteins are needed to explore their structural properties and multifunctionality by testing a wider range of proteins. Conjugation with functional groups that induce multi-stimuli responsiveness for controllable release is another area that is necessary to be considered. Preclinical results and in vivo tests will be useful for self-assembled and ionically crosslinked systems. Combination of electrostatic and chemical crosslinking should be explored to balance the requirement for structural stability and biocompatibility. NGs and MGs with regards to their preparation method, stimuli-responsiveness and potential for scale-up of manufacturing process can lead in the future to novel and stable products of enhanced quality and efficiency.

## Figures and Tables

**Table 1:** Examples of MGs/NGs as oil-in-water emulsion stabilizers.

Polysaccharide	Size	Formation Mechanism	Ref.
Chitosan	NG	Amide bonding with stearic acid (EDC-mediated reaction)	[18]
	NG	Crosslinking with genipin	[19]
	MG	Crosslinking with genipin	[20]
Chitosan/carboxymethyl starch	NG	Amide bonding with stearic acid (EDC-mediated reaction)	[21]
Chitosan/Myristic acid	NG	Amide bonding(EDC-mediated reaction)	[22]
Whey protein/dextran	MG	Maillard conjugation, top-down method	[23]
Pectin	MG	Crosslinking with CaCl_2_	[24]
Pectin	MG	Crosslinking with CaCl_2_	[25]
Cyclodextrin	NG	Crosslinking with 1,4-phenylene diisocyanate (PDI)	[26]

**Table 2:** Examples of MGs/NGs as encapsulation carriers of nutraceuticals.

Polysaccharide	Size	Crosslinking Method	Loaded Substance	Potential for Applications	Ref.
Alginate	MG	Emulsification/internal gelation, Polyelectrolyte complexes (Chitosan-Chondroitin sulfate)	Anthocyanins	Controlled delivery	[44]
	MG	CaCl_2_ crosslinking	Rutin, tiliroside, β-carotene, curcumin	Therapeutic	[45]
Pectin	NG	Ionic gelation with sodium tripolyphosphate	Resveratrol	Ocular treatments	[46]
	NG	Covalent bonding with citrate	Green tea	Antioxidant activity	[47]
	MG	Ionotropic gelation, CaCl_2_ crosslinking	*Lactobacillus casei/rhamnosus*	Storage stability enhancement, protected delivery	[48]
Carboxymethyl konjac glucomannan/chitosan	NG	Covalent bonding, electrostatic interactions, EDC/NHS crosslinking	Curcumin	Controlled release	[49]
Soy polysaccharide	NG	Self-assembly with Soy protein	Folic acid	Controlled release, protected delivery	[50]
Hyaluronic acid	MG	Enzymatic crosslinking of tyramine conjugated HA in the presence of HRP and H_2_O_2_	Lysozyme, TGF-β1	Sustained release	[51]
Chitosan	NG	Ionic gelation with sodium tripolyphosphate	Fucoxanthin	Controlled release, Storage stability enhancement	[52]
Chit/Alginate	MG	Electrostatic interactions	*Juglans regia* L. polyphenols	Sustained release	[53]

**Table 3:** Examples of polysaccharide NGs with applications in medical sciences.

Polysaccharide	Formation Mechanism	Loaded Substance	Potential for Applications	Ref.
Hyaluronic acid	crosslinking by glycerol diglycidyl ether in emulsion	3 ((E) 3 (4 hydroxyphenyl)acryloyl) 2H chromen 2 one	Blood compatibility, loading and release in biological fluids	[65]
	electrostatic complexation with keratin and crosslinking by peroxide	DOX	Antitumor activity	[66]
	Self-assembled by DEGMA side chains and coumarin	paclitaxel	Activity against ovarian cancer cells	[67]
	Disulfide bonds by methacrylating with cystamine	Cationic DOX	Glioma therapy	[68]
	electrostatic complexation with poly-l-lysine	GFP, DOX and VAN	chemotherapy and antibiotic activity	[69]
	radical polymerization of methacrylated hyaluronic acid with cystamine bisacrylamide	DOX and Au nanoclusters	tumor cell inhibition	[70]
	electrostatic complexation with Fbg and thermal treatment	Curcumin	therapeutic and diagnostic	[71]
Chondroitin sulfate	Self-assembling grafted by octadecylamine	Curcumin	Activity against human breast cancer cells	[72]
	Self-assembly by conjugated prednisolone	Prednisolone	treatment of ulcerative colitis	[73]
	Self-assembly by conjugated prednisolone	Prednisolone	Treatment of rheumatoid arthritis	[74]
	self-assembly by conjugated methotrexate	Methotrexate	activity against A549T and Hela tumor cells	[75]
	electrostatic complexation with BSA and thermal treatment	β-Carotene	Therapeutic	[76]
Chitosan	grafted phenylamine for host-guest interaction in the presence cucurbit[8]uril	DOX	Hindering the growth of human lung cancer cells	[77]
	Ionic gelation with tripolyphosphate	Honey	Effect of laponite on drug loading/release	[78]
	Ionic gelation with tripolyphosphate and enzymatic by peroxide	5-Fluorouracil	Effect of crosslinking and pH conditions on drug release	[79]
Alginate	Crosslinking by CaCl_2_	Ovalbumin	Dendritic cell targeting	[80]
	Self-assembly by conjugated prednisolone	Prednisolone	Arthritis therapy	[81,82]
	ionic crosslinking with gadolinium in reverse microemulsion	Hydrophilic drugs and rhodamine b	Treatment of neurodegenerative diseases and MRI	[83]
	disulfide modification and CaCl_2_ crosslinking	DOX and superparamagnetic iron oxide NPs	chemotherapy and MRI	[84]
	CaCl_2_ crosslinking	DOX and GL	treatment of hepatocellular carcinoma	[85]
Pullulan	Physical crosslinking by conjugated cholesterol units	Insulin	Tongue muscle regeneration	[86]
	crosslinked with terminal thiol group polyethylene glycol	Human dermal fibroblasts/osteoblasts	Osteogenic NG transplants	[87]
	Physical crosslinking by conjugated cholesterol units	Ovalbumin	Anticancer immunotherapy	[88]
	electrostatic complexation with fucoidan and genipin crosslinking	miRNA	Atherothrombosis treatment	[89]
κ-Carrageenan/Chitosan	copolymerization of acrylamide and sodium acrylate	Rivastigmine	Biocompatibility, drug release, incorporation of carbon dots	[90]
carboxymethyl cellulose	electrostatic complexation with BSA and thermal treatment	Camptothecin, ^132^I	Therapeutic and diagnostic action	[91]
Xanthan gum	electrostatic complexation with BSA and thermal treatment	Curcumin	Therapeutic	[92]
Dextran	thermal treatment with lysozyme	DOX and Au NPs	Optical cell imaging and cancer treatment	[93]

**Table 4:** Examples of polysaccharide MGs with applications in medical sciences.

Polysaccharide	Formation Mechanism	Loaded Substance	Potential for Applications	Ref.
Hyaluronic acid	Fe^3+^ and Gd^3+^ crosslinking in reverse micelle emulsion medium	Fe and Gd	Blood contacting applications and MRI signal enhancers	[94]
	Crosslinikng by divinyl sulfone	VAN	Sustainable drug delivery	[95]
Alginate/Chondroitin sulfate/Silk fibroin	Droplet microfluidics and Ca^2+^/Zn^2+^ crosslinking	PS NPs and BSA-coated PS NPs	Controlled release of drug-loaded NPs	[96]
Chitosan	Schiff-base crosslinking reaction	BSA and AgSD	Wound dressings	[97]
	Schiff-base crosslinking reaction	BSA	Pulmonary drug delivery	[98]
	Emulsion polymerization	vitamin-B12	Oral drug delivery	[99]
Alginate	microspheres crosslinking by CaCl_2_	RPMI 8226 cells	Model for drug resistance in multiple myeloma	[100]
	metal ions crosslinked hydrogel and microfluidic preparation	mesenchymal stem cells	Microenvironment for osteogenesis	[101]
	chemically bonded interpenetrating hydrogels with HA and squeezing	Human umbilical vein/mouse aortic endothelial cells	Tissue regeneration	[102]
	crosslinking by CaCl_2_ in a microfluidic device	Bone marrow mesenchymal stem cells	Scaffolds for bone regeneration	[103]
Dextran	Schiff base reaction with diamine in w/o inverse emulsion	magnetic NPs and DOX	DOX release	[104]

## Data Availability

The data presented in this study are available on request from the corresponding author.
